# Ecohydrological effects of water conveyance in a disconnected river in an arid inland river basin

**DOI:** 10.1038/s41598-022-14524-z

**Published:** 2022-06-15

**Authors:** Yaning Chen, Yapeng Chen, Chenggang Zhu, Yang Wang, Xingming Hao

**Affiliations:** 1 State Key Laboratory of Desert and Oasis Ecology, Xinjiang Institute of Ecology and Geography, Chinese Academy of Sciences, Urumqi, 830011 China; 2 College of Grassland and Environmental Sciences, Xinjiang Agricultural University, Urumqi, 830052 China; 3 Akesu National Station of Observation and Research for Oasis Agro-Ecosystem, Akesu, 843017 China

**Keywords:** Ecology, Hydrology

## Abstract

Water system management is a worldwide challenge, especially in arid and semi-arid regions. Ecological water conveyance projects aim to raise the groundwater table, thereby saving natural vegetation and curbing ecological deterioration. Since 2000, these projects have been implemented in the arid zone of northwest China, with generally successful outcomes. Taking a portion of the lower reaches of the Tarim River as the study area, this paper analyzes in detail the ecohydrological effects which have occurred since the launching of artificial water conveyance 20 years ago. The results show that the groundwater table in the upper, middle and lower segments of the Tarim River’s lower reaches has been raised on average 4.06, 4.83 and 5.13 m, respectively, while the area of surface water bodies connected to those sections has expanded from 49.00 km^2^ to 498.54 km^2^. At the same time, Taitema Lake, which is the terminal lake of the Tarim River, has been revived and now boasts a water area of 455.27 km^2^. Other findings indicate that the surface ecological response is extremely sensitive and that the area of natural vegetation has expanded to 1423 km^2^. Furthermore, the vegetation coverage, vegetation index (NDVI), and Net Primary Productivity (NPP) have increased by 132 km^2^, 0.07 and 7.6 g C m^−2^, respectively, and the Simpson dominance, McIntosh evenness, and Margalef richness indices have risen by 0.33, 0.35 and 0.49, respectively, in the monitored sample sites. As well, the carbon sink area has expanded from 1.54% to 7.8%. Given the increasing intensity of the occurrence of extreme hydrological events and successive dry years, similar ecological water conveyance projects should be considered elsewhere in China and in other parts of the world. The water conveyance scheme has generally proven successful and should be optimized to enhance the benefits of ecological water conveyance under water resource constraints.

## Introduction

In arid zones, water resources are scarce, unpredictable, unstable, and ecologically fragile. The relative abundance of natural resources and the extreme fragility and instability of the ecological environment are intertwined, so that the contradiction between ecological maintenance and economic development can be challenging^1^. Over the past half-century, the agricultural production and socio-economics of the arid zone oasis in China’s vast northwest have been intensively developed, while at the same time the ecological environment has undergone enormous changes. These changes have manifested as massive water diversion in the middle and upper reaches of rivers, the expansion of artificial oases, and the enhancement of land productivity. Meanwhile, and often as a direct result of these interventions, downstream rivers have broken into dry streams, terminal lakes have disappeared, broad expanses of natural vegetation have withered and died^2^, and desertification intensity has increased.

In response to this situation, the Chinese government launched a series of ecological water conveyance projects in 2000, aiming to salvage and restore the ecology of some of the larger inland rivers in the country’s Northwest Arid Zone^3,4^. For example, the Tarim River in Xinjiang, which is China’s largest inland river, was cut off 321 km downstream of the reservoir following the construction of the Daxihai Reservoir in 1972. As a result, its terminal, Taitema Lake, eventually dried up^5^. However, thanks to the ecological water conveyance, the lake has since been revived through the transfer of 84.45 × 10^8^ m^3^^6^ of water (21 transfers in total) over the course of 20 years.

The Heihe River, located in the Hexi Corridor, suffered a fate similar to that of the Tarim. The Heihe is the second largest inland river in China and originates in the Qilian Mountains. From the mid-1960s to the beginning of the twenty-first century, 58 reservoirs were built along the Heihe River, of which 40 were in the plains. The large-scale water conservancy construction and high-intensity water resources development resulted in the river being broken downstream and its terminal, the Juyan Lake, drying up by the 1990s^7^. In response to these devastating changes, ecological water conveyance was implemented in the Shiyang River in the eastern Hexi Corridor and the Konqi River in the northeastern Tarim Basin^8,9^.

The study of ecological water conveyance is a growing field. For example, over the past few decades, numerous scholars have studied issues such as groundwater level and water quality changes^10–12^, the bonding relationship between groundwater system and vegetation^5,13^, water uptake capacity and characteristics of plant roots^14^, hydraulic uplift and water redistribution of plant roots^15,16^, and the physiological and ecological response processes of surface vegetation^17,18^. The aim of these studies has been to analyze groundwater changes and surface ecological responses during water conveyance. There are also useful studies on how to expand ecological water conveyance benefits, ecological water demand, and ecohydrological processes and mechanisms^19–21^. All of these studies have effectively improved the scholarship of ecohydrology and promoted the development of ecohydrology in arid regions.

In the present paper, we examine changes in groundwater table depth and the response of surface ecological processes after the implementation of the water conveyance projects. In so doing, we take into account the monitoring results of ecological water conveyance in the lower reaches of the Tarim River over the past 20 years. We also make suggestions to further expand the ecological benefits of water conveyance, aiming to provide appropriate technological support for the scientific scheduling of this process.

## Results and discussion

The water table depth, surface water body area, and surface ecological processes have all changed significantly during the 20 years the ecological water conveyance projects have been underway in the lower reaches of the Tarim River. Specifically, there has been a notable increase in the water table, surface water body area, vegetation density and coverage, the vegetation index (NDVI), Net Primary Production (NPP) of natural vegetation, and ecosystem function and health. The following sections provide details on these changes.

### Changes in groundwater table depth

**Figure 1:**
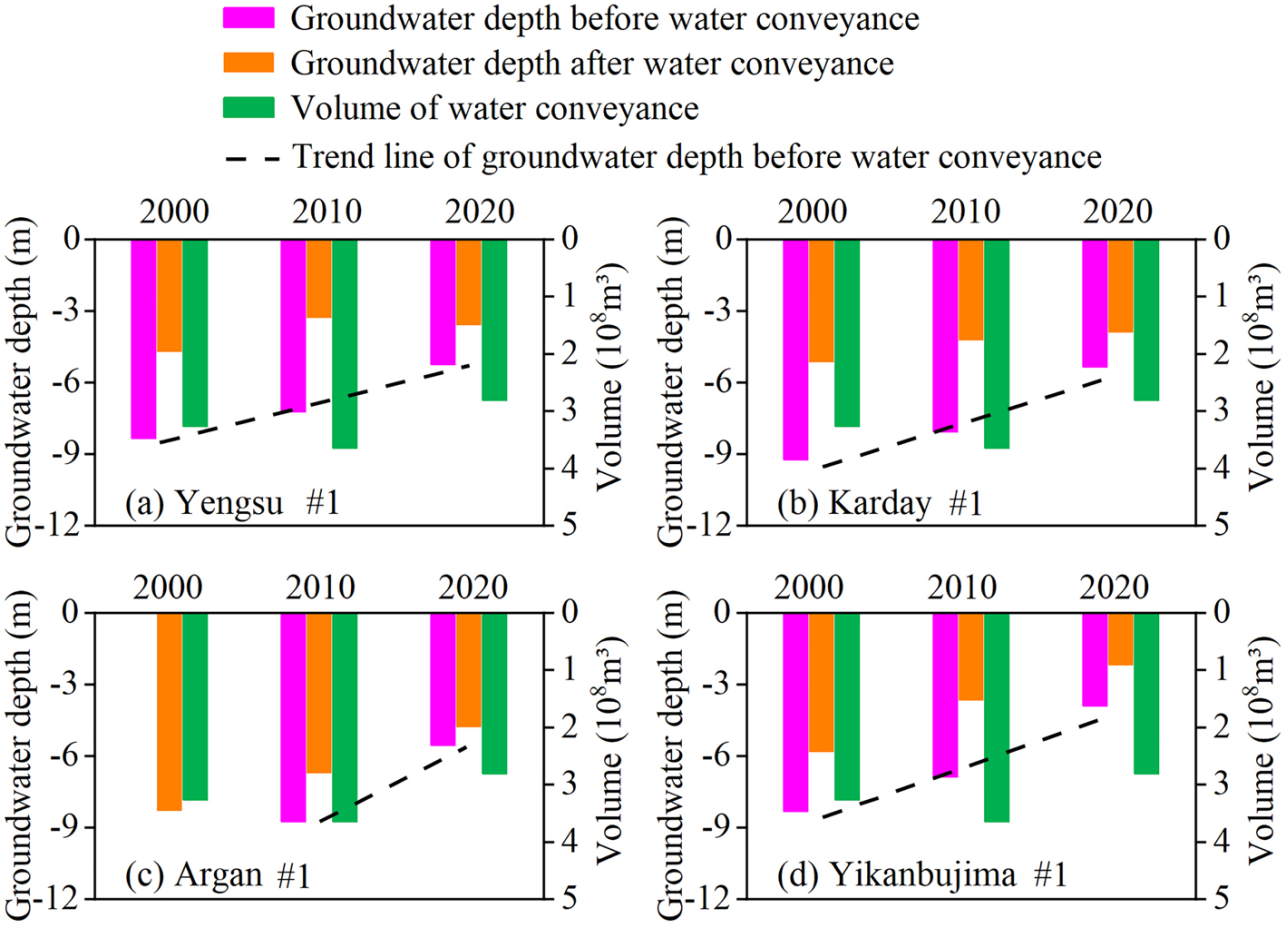
Changes in groundwater depth of typical monitoring cross-sections pre- and post-conveyance of water in the lower reaches of Tarim River from 2000 to 2020. Yengsu, Karday, Argan and Yikanbujima are four monitoring sections in the lower reaches of Tarim River. “#1”is the No. 1 groundwater level monitoring well on each monitoring section, which is located 50 m away from the river.

Groundwater (soil water) is the most important water source for maintaining natural vegetation in the lower reaches of the Tarim River, as the climate is extremely arid and atmospheric precipitation has little ecological significance. The changes in water table depth are directly related to the composition, distribution, and growth of the natural vegetation of the desert riparian forest, which in this case is mainly *P. euphratica*^5^. During the past 20 years, the ecological water conveyance in the lower reaches of the Tarim has been intermittent, and the groundwater table elevation has been closely related to the water conveyance. From the analysis of the groundwater table’s rise in the upper, middle, and lower reaches of the Tarim River (Fig. 1), the magnitude of the uplift is clearly related to four crucial factors: the groundwater table depth prior to the water conveyance, the volume of water discharge, the duration of the transfer, and the water head location.

In the early stages of the water conveyance projects (2000–2010), the groundwater table in the upper and middle segments of the lower reaches of the Tarim River rose to a relatively large extent, while the groundwater table in the lower segment of the river only showed an increasing rising trend after 2011. The monitoring results reveal that after nearly 20 years of ecological water conveyance, the groundwater table in three sections of the lower reaches of the Tarim has been affected at a range of more than 1000 m. The three sections are the Yengsu section in the upper segment, the Karday section in the middle segment, and the Yiganbujima section in the lower segment. Furthermore, the groundwater table has risen by 2.69, 1.38 and 1.59 m, respectively, in these three sections^22^. Within 100 m from the river, the water table depth rose from 7.76, 9.31 and 7.82 m prior to ecological water conveyance to 3.70, 4.48, and 2.69 m, and 4.06, 4.83, and 5.13 m, respectively, after it. Within 500 m from the river, the water table rose by 1.6, 3.99, and 5.26 m, respectively. The shallow groundwater in the lower reaches of the Tarim River has also been recharged to a certain extent, and the lateral influence range is still gradually expanding.

### Changes in water body area

**Figure 2:**
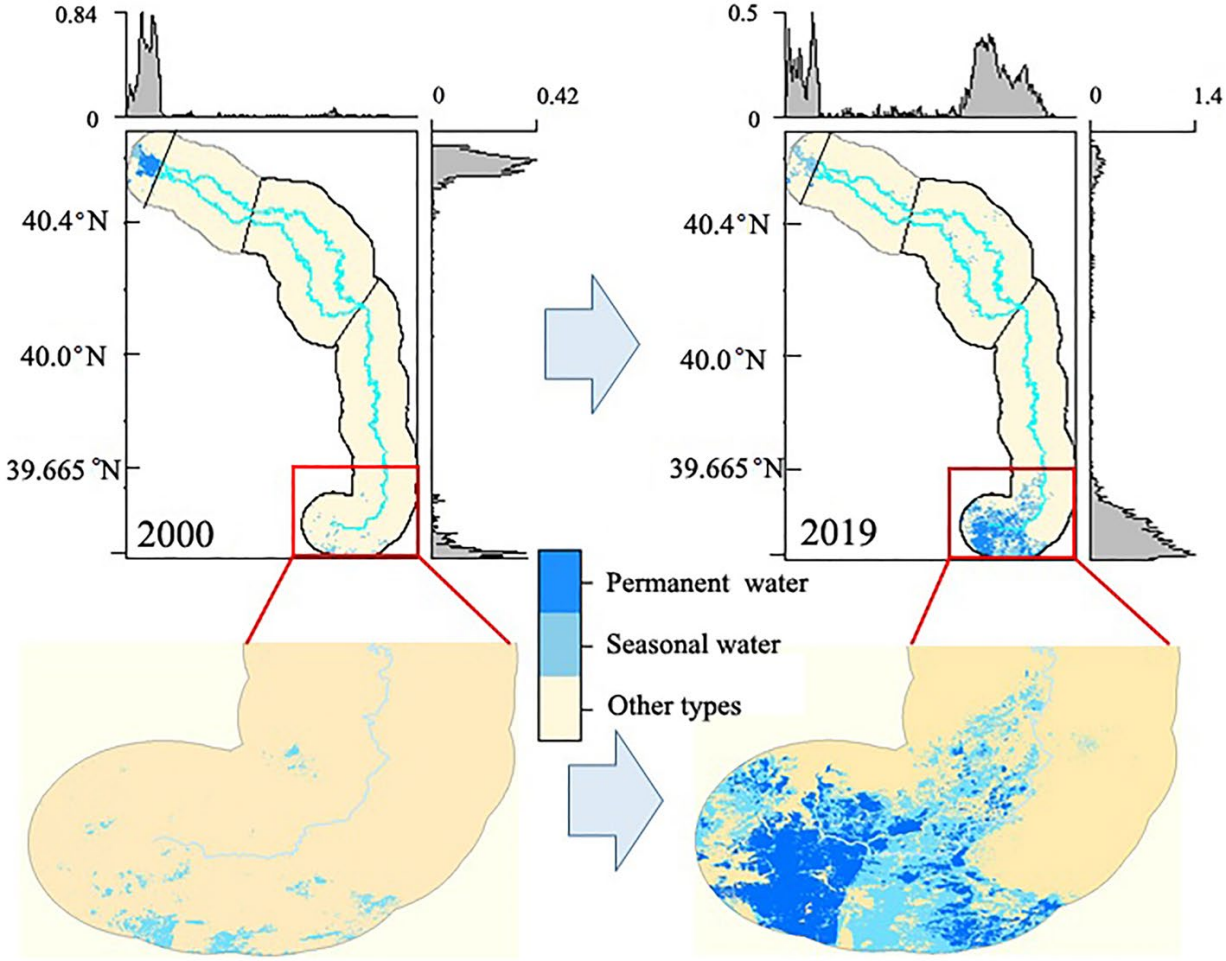
Spatial distribution of water surface area in lower reaches of Tarim River in 2000 and 2019. The subfigures were generated in R 4.0.2 (https://cran.r-project.org/bin/windows/), and then merged in Microsoft PowerPoint 2013 (https://www.microsoft.com/).

The changes in water body area in the lower reaches of the Tarim River are closely related to the amount of water delivered via conveyance. During the past 20 years, the surface water body area, seasonal water body area, and permanent water body area all decreased to the lowest point in 2009, with the river water failing to reach Taitema Lake, the river's terminal, in 2006, 2007, and 2009^23^. The surface water body area, seasonal water body area and permanent water body area in the river’s lower reaches fluctuated and increased during the ecological water conveyance process. In particular, the seasonal water body area in the upstream section showed a significant expansion. The area increase rate of surface water, seasonal water, and permanent water in the middle section from Yengsu to Argan is 1.75 km^2^ a^−1^, 1.58 km^2^ a^−1^, and 0.16 km^2^ a^−1^, respectively. Similarly, the area of surface water bodies, seasonal water bodies, and permanent water bodies in the lower section (below Argan) increased at the rate of 13.48 km^2^ a^−1^, 8.24 km^2^ a^−1^, and 5.23 km^2^ a^−1^, respectively. It is worth mentioning that the area of surface permanent water body and seasonal water body in Taitema Lake significantly increased, with the area of the lake waters expanding 417.08 km^2^, from 38.19 km^2^ in 2000 to 455.27 km^2^ in 2019. This represents a nearly 12-fold increase (Fig. 2).

### Vegetation sample site monitoring analysis

The vegetation species in the lower reaches of the Tarim River were sparsely distributed, with *P. euphratica* and *Tamarix* sp. as the main established species. In the longitudinal direction, surface vegetation coverage and species number decreased as the water table depth increased from the upper and middle segments to the lower segment. In the lateral direction, surface vegetation shows the same trend, with groundwater table depth increasing the greater the distance from the river^13^.

The surface ecological processes in the lower reaches of the Tarim River have responded positively to the water conveyance project, with density, coverage and the number and diversity of species significantly increasing. However, the response of surface ecological processes to the changes in groundwater table uplift has varied from section to section. In the lateral direction, the groundwater table in areas nearer to the river had a more prominent rise and the response of surface vegetation was stronger, whereas the groundwater table rise in areas farther from the river was smaller and so the response of surface vegetation was weaker. In the longitudinal direction, the same trend was observed from the upper to the lower segments in response to changes in the groundwater table. In this paper, we analyze the changes in detail by taking a closer look at the Yengsu section, which is located at the beginning of the middle section of the lower reaches of the Tarim River. In so doing, we apply sample site investigation and dynamic monitoring of the groundwater table to the study area.

#### Changes in vegetation density and coverage

**Figure 3:**
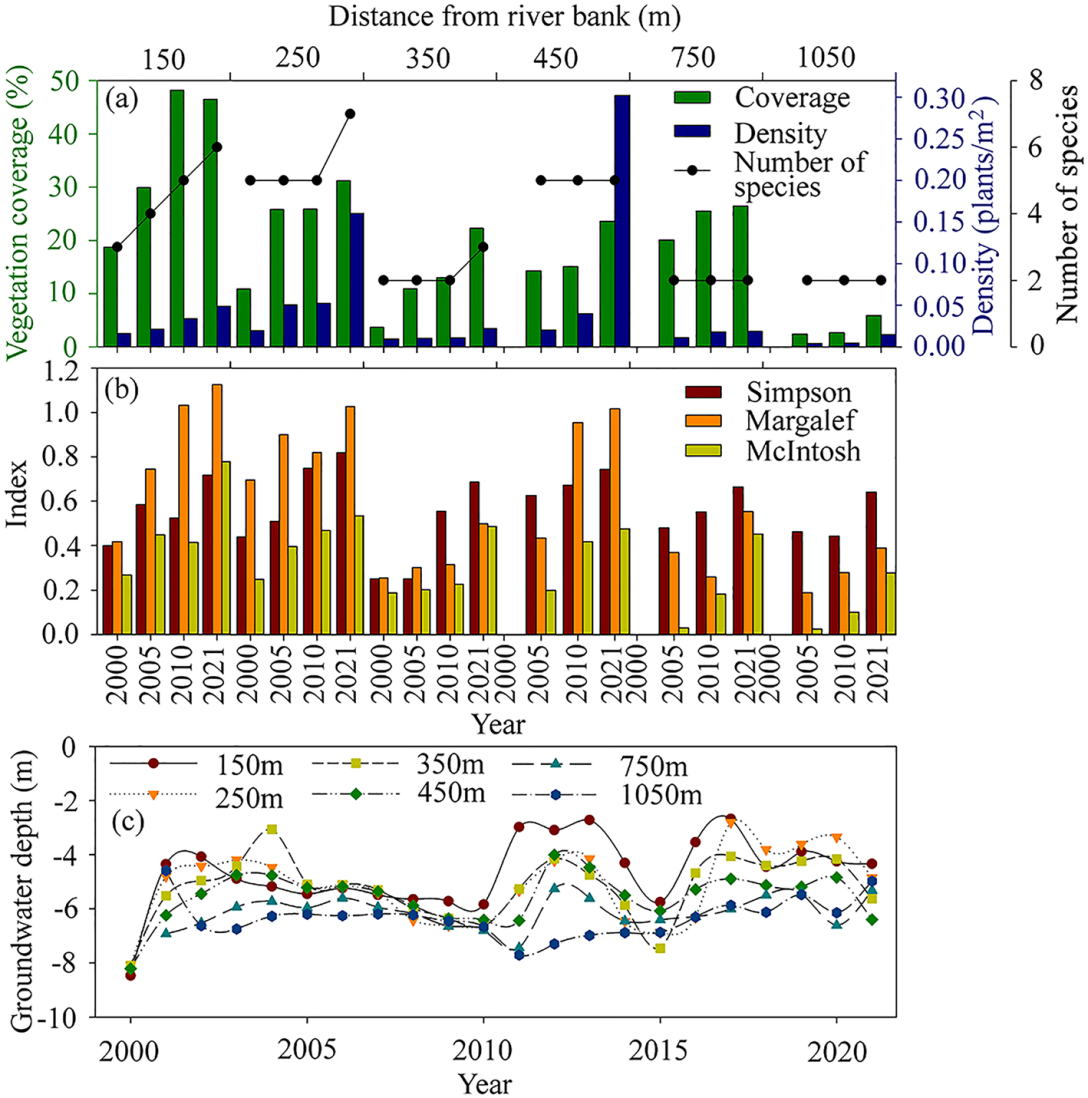
Changes in vegetation coverage, density and number of species (**a**), species diversity indices (**b**), and groundwater depth (**c**) for each site at Yengsu section in the lower reaches of Tarim River.

The results of our sample site monitoring show notable positive changes in groundwater depth between 2000 and 2021 as a direct result of the ecological water conveyance initiative. At 150 m from the river, the groundwater table depth rose from 8.47 m to 4.34 m, respectively, representing an uplift of 4.13 m (Fig. 3c). Moreover, the vegetation coverage and density increased from 18.77% and 0.016 plants/m^2^ to 46.51% and 0.049 plants/m^2^, and the number of species doubled from three to six.

At 250 m from the river, the groundwater table depth rose from 8.07 m in 2000 to 4.85 m in 2021, representing an uplift of 3.22 m. The vegetation coverage and density increased from 10.89% and 0.020 plants/m^2^ to 31.24% and 0.160 plants/m^2^, respectively, and the number of species jumped from five to seven.

At 350 m from the river, the water table rose 2.48 m between 2000 and 2021. The vegetation coverage and density increased from 3.69% and 0.010 plants/m^2^ to 22.27% and 0.022 plants/m^2^, respectively, and the number of species increased from two to three. It is worth noting that the expansion in vegetation cover in the first three sample sites was mainly due to the increase in the number and canopy width of herbs and shrubs that occurred as a direct result of the ecological water conveyance process.

At 750 m from the river, the groundwater table depth rose from 5.96 m to 4.98 m between 2005 and 2021, respectively, representing an uplift of 0.64 m, while the vegetation coverage and density increased from 20.07% and 0.011 plants/m^2^ to 26.43% and 0.019 plants/m^2^, respectively.

At 1050 m from the river, the sample site had an elevated water table of 1.22 m. The vegetation coverage and density increased from 2.41% and 0.004 plants/m^2^ in 2005 to 5.89% and 0.0148 plants/m^2^ in 2021, respectively (Fig. 3a). Among them, the increase in canopy area of *Tamarix* sp*.* and *P. euphratica* in the sample site was the main reason for the expansion in coverage.

#### Changes in species diversity indices

Plant richness and evenness in the lower reaches of the Tarim River were low, with species diversity indices showing significant changes in response to the ecological water conveyance and the rise in the groundwater table (Fig. 3b). For example, at the Yengsu section, the Simpson dominance index, McIntosh evenness index and Margalef richness index, which reflect changes in species diversity, decreased from 0.58, 0.45 and 0.74 in 2005 to 0.46, 0.03 and 0.03, respectively. These changes occurred in response to the increase in groundwater depth from the first sample site at 150 m to the sixth sample site at 1050 m from the river channel. After 20 years of ecological water conveyance, the Simpson dominance index, McIntosh evenness index and Margalef richness index had increased on average by 0.33, 0.35 and 0.49, respectively, in the first three sample sites (Fig. 3b).

### Vegetation index (NDVI) changes

The Normalized Difference Vegetation Index (NDVI) is an important indicator of vegetation growth^24^. The study results reveal that the NDVI of the lower reaches of the Tarim River increased from 0.14 in 2000 to 0.21 in 2020, representing a rise of about 33.3%. The ecological water conveyance expanded the river region’s natural vegetation 188%, from 492 km^2^ in 2000 to 1423 km^2^ in 2020. Specifically, the area of low, medium, and high vegetation cover expanded by 277 km^2^, 537 km^2^ and 132 km^2^, representing increases of 20.8%, 448% and 190%, respectively. Further analysis of changes in vegetation coverage at different river sections indicate that the area of low vegetation coverage in the upper and middle segments showed a decreasing trend, whereas the area of medium and high vegetation coverage in the upper and middle segments showed an increasing trend. This latter trend was especially prominent in the middle segment, where the increase in the area covered by medium and high vegetation was relatively large.

**Figure 4:**
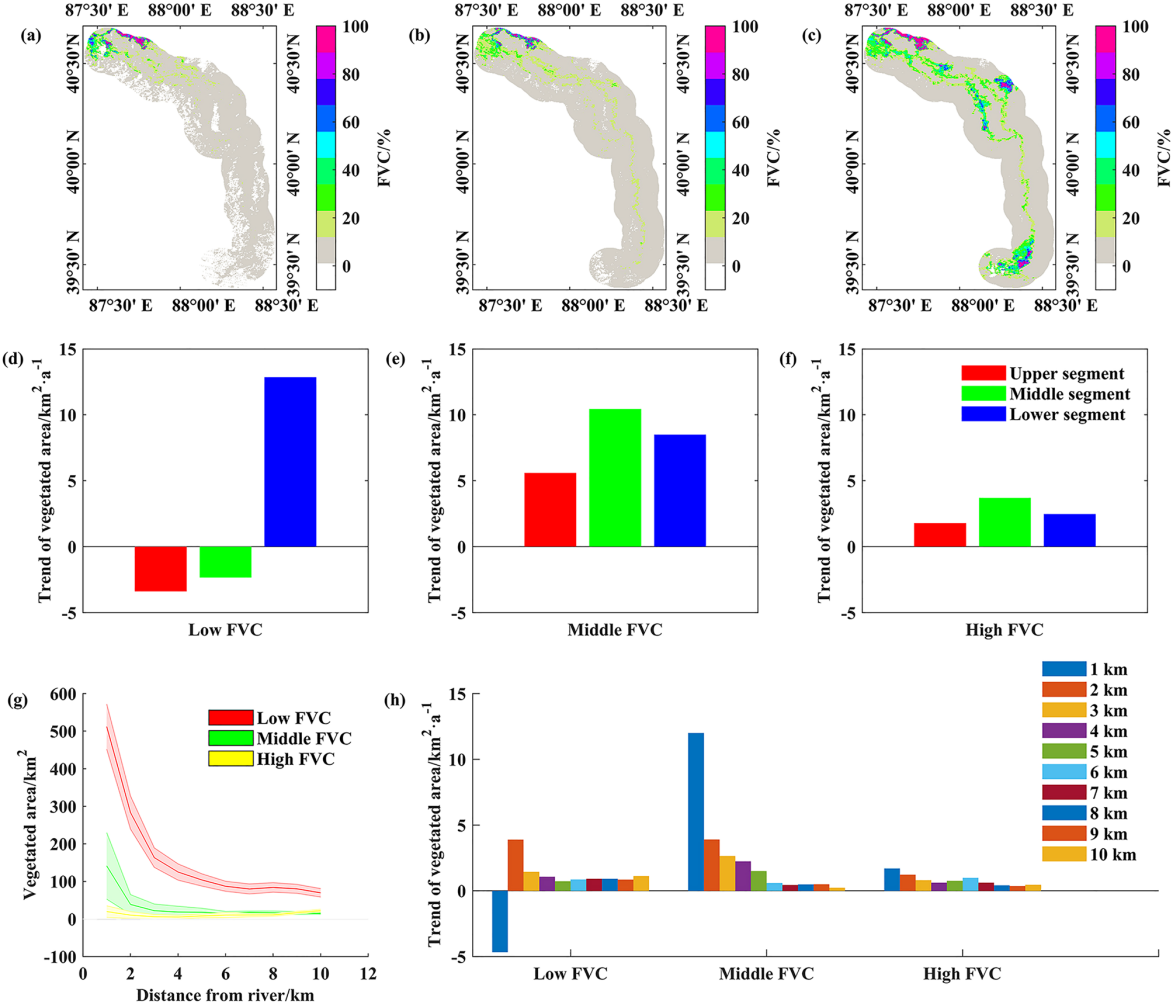
Variation of vegetation cover in the lower reaches of Tarim River. Spatial distribution of fraction of vegetation cover in (**a**) 2000, (**b**) 2010 and (**c**) 2020. Trends of (**d**) high fraction of vegetation cover, (**e**) middle fraction of vegetation cover and (**f**) low fraction of vegetation cover in different river sections. (**g**) Vegetation area and (**h**) change trend at different distances from the river.

In the downstream segment, the area covered by all types of vegetation showed an upward trend, with the area covered by low vegetation expanding significantly (Fig. 4). In the lateral direction, the NDVI within 2 km of the water conveyance channel showed a more obvious response with greater increases, while NDVI beyond 2 km from the channel revealed smaller increases^25^. These differences reflect the influence range of the ecological water conveyance.

### Changes in net primary production (NPP) of natural vegetation

Net primary production (NPP) is a key parameter of carbon cycling and energy flow in terrestrial ecosystems. NPP not only reflects terrestrial ecosystem productivity, but also characterizes the quality of terrestrial ecosystems and plays an important role in global change and carbon balance^26,27^. The results of our study show that the area of natural vegetation in the lower reaches of the Tarim River with highly significant and significant increases in NPP during the study period accounted for 31.93% (P < 0.01) and 11.49% (P < 0.05), respectively, with an increase rate of 0.40 g C·m^−2^·a^−1^. The increases were greater in the upper and middle segments of the lower reaches of the river than in the lower segment. In terms of vegetation type, the magnitude of the multi-year mean NPP was in the order of *Tamarix* spp. community > *P. euphratica* community > herbaceous community. The largest increase in NPP was observed in the *Tamarix* spp. community, rising 350.20% from 2001 to 2019^28^.

### Area changes in vegetation carbon sink area

The ecological water conveyance project in the lower reaches of the Tarim expanded the vegetation coverage and enhanced the carbon sequestration capacity of the region through photosynthesis. The lower reaches of the river are dominated by desert and sparse vegetation, and the ecosystem carbon sinks are mainly low carbon sinks. The monitoring results of the study show that the vegetation carbon sink area in the river’s lower reaches indicate a gradual expansion under the influence of the ecological water conveyance^29^, increasing from 1.54% of the study area in 2001 to 7.8% in 2020. As well, the Net Ecosystem Productivity (NEP) of the area’s vegetation showed an increasing trend at a rate of 0.541 g C·m^−2^·a^−1^, with the largest increase – 0.406 g C·m^−2^·a^−1^ – occurring in summer^29^and no significant carbon sink area in winter.

**Figure 5:**
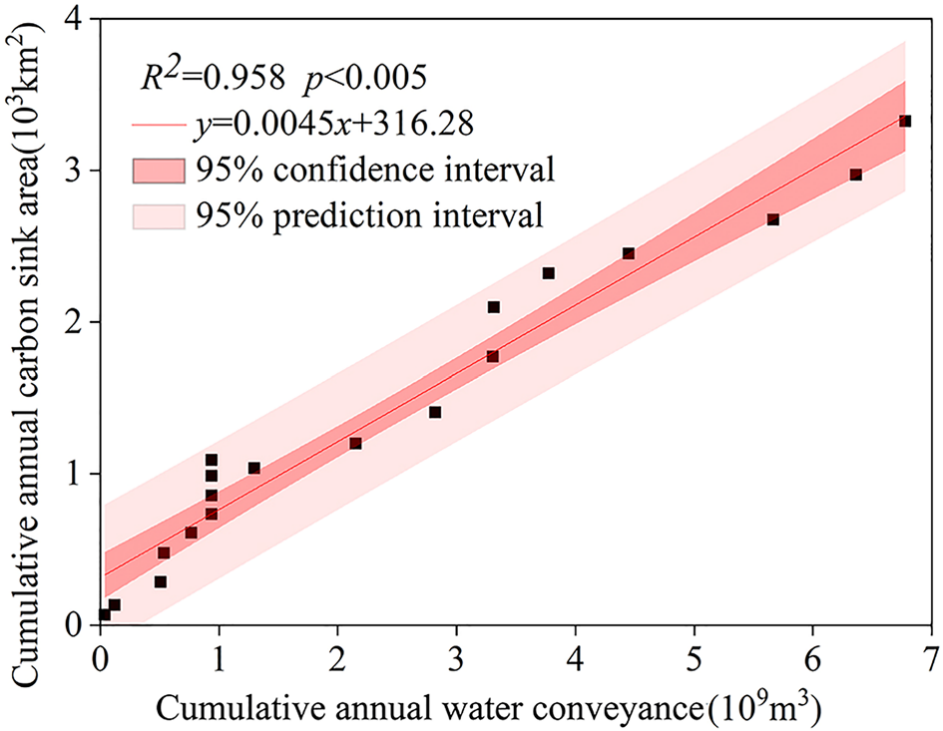
Linear fitting chart of cumulative water conveyance and cumulative carbon sink in the lower reaches of Tarim River.

Furthermore, in order to quantitatively investigate the degree of influence of ecological water conveyance on the carbon sink area in the lower reaches of the Tarim, a linear fit of cumulative water conveyance and carbon sink area was performed (Fig. 5). Based on the results, a strong linear correlation was found between cumulative water conveyance and carbon sink area (R^2^ = 0.958, *p* < 0.005). The data points were all within the 95% confidence interval, indicating that, with the increase of ecological water conveyance in the lower reaches of the river, the carbon sink area appeared to respond positively, taking into account a lag effect of about one year.

## Conclusion

River cutoff and ecological degradation in the lower reaches of inland river basins in arid regions is a growing problem. In order to salvage and restore damaged ecosystems of disconnected rivers, ecological water conveyance projects were implemented in several watersheds in the arid zone of northwest China, starting in 2000. Since then, significant ecological benefits have been achieved. For example, in the Tarim River’s lower reaches, the groundwater table on both sides has risen by 3–5 m, and the range of influence exceeds 1000 m from the river. Additionally, the response range of natural vegetation has expanded from 492 km^2^ to 1423 km^2^, and the river's terminal, Taitema Lake, has been revived, forming a water area of 455.27 km^2^. These results clearly indicate that the continuous ecological deterioration along the banks of the lower reaches of the Tarim River has successfully been curbed.

However, because the conveyance projects have mainly taken the form of natural drainage along the river channel, the ecological response has been slow and the impact relatively limited. Only the groundwater table near both sides of the river has effectively been raised. The three main manifestations of this process in the upper and middle segments of the lower reaches of the Tarim River are as follows: (1) The analysis of changes in groundwater level shows that the recent groundwater table rise has slowed significantly and has tended to reach an equilibrium state at some sections. Therefore, the intensity of the impact of the artificially introduced water on surface vegetation is weakening. (2) The natural water conveyance method along the river channel cannot achieve the purpose of plant seeding and regeneration. Further, this method will likely not help the region obtain ecological sustainability, as it is even more difficult to make a large and rapid recovery of the growth of grass and shrub vegetation on both sides of the riverbank. (3) A large amount of river water has flowed into the terminal lake (Taitema Lake). Although this has led to an expansion of the water area, most of the water bodies are being dissipated by evaporation.

Given the above challenges, we suggest taking the following course of action to remedy them. (1) The water conveyance currently proceeds on a “linear” path along the natural river to maintain the rivers ecological flow. However, the subdivision of surface overflow water supply from top to bottom could be engineered to promote the seeding and regeneration of *P. euphratica*, *Tamarix* spp. and other species, as well as the recovery of grass and shrub vegetation on both sides of the riverbank. (2) In areas with soil characterized as seed-poor, river overflow could help disperse artificial floating seeds to promote the growth of surface vegetation. This would rapidly increase the surface coverage and expand the scope and effect of the ecological water conveyance area. (3) Taitema Lake needs to be scientifically assessed and its ecological function and suitable water area determined. At the same time, the uncertainty brought by the increase in frequency and intensity of extreme hydrological events caused by global climate change and consecutive dry years needs to be studied in relation to ecological water conveyance and ecological restoration.

## Materials and methods

### Study area

**Figure 6:**
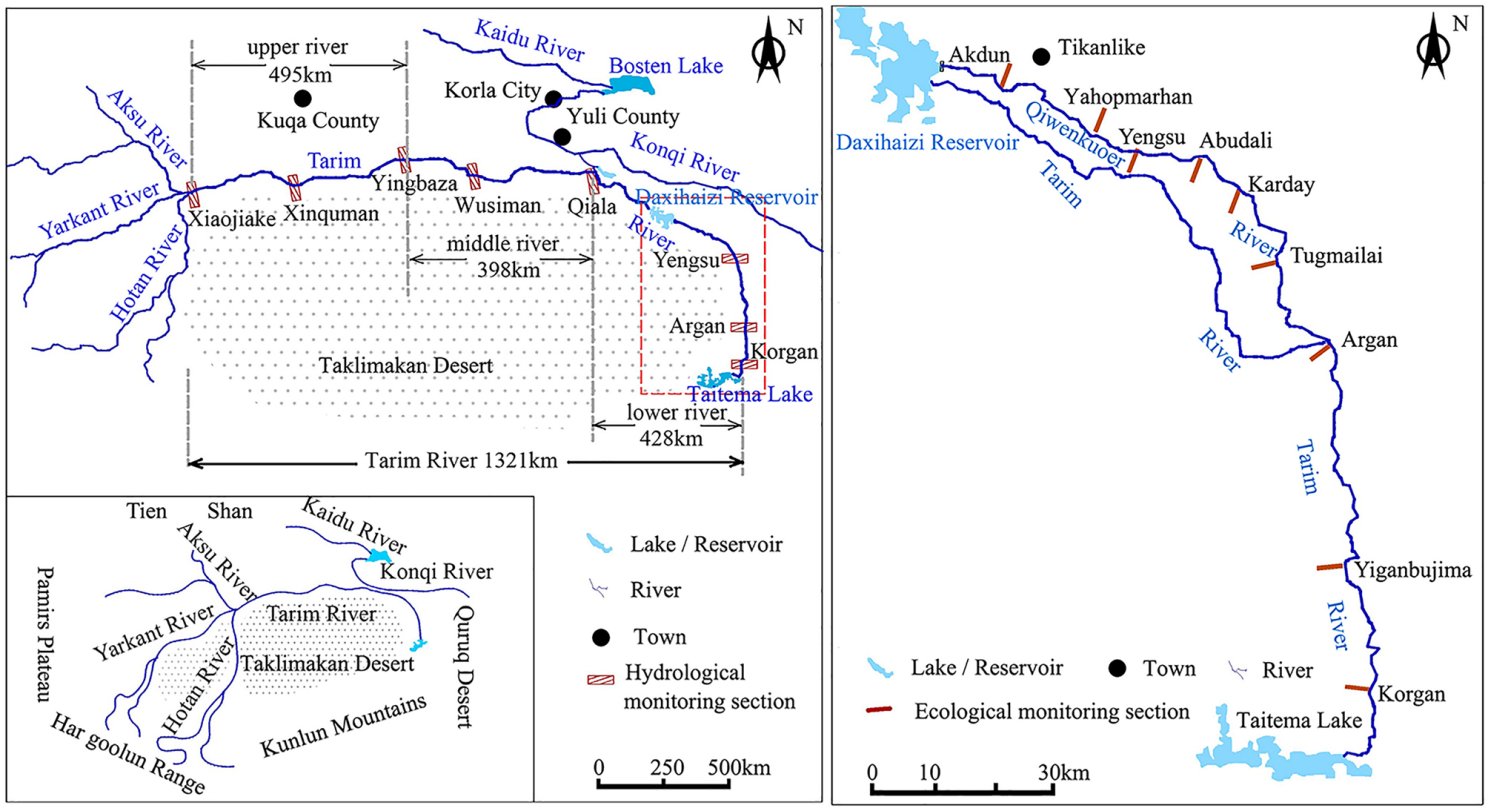
Distribution map of the lower reaches of Tarim River. This map was constructed using ArcGIS 10.3 (http://www.esri.com/software/arcgis/arcgis-for-desktop).

The Tarim River Basin is located in the south of Xinjiang, China, with the Tianshan Mountains to the north, the Kunlun Mountains to the south, and the Pamirs Plateau to the west. It consists of 9 major headwaters and 114 water systems, covering an area of about 102 × 10^4^ km^2^. Due in part to its inland remote positioning, the basin is characterized by the duality of relatively abundant natural resources and an extremely fragile ecological environment. The main stream of the Tarim River is 1,321 km long and is roughly divided into the upper, middle and lower reaches (Fig. 6), the latter which is located between the Taklamakan Desert and the Quruq Desert, with the Qiala hydrological station as the connector node. The terminal of the Tarim River is Taitema Lake. The lower reaches of the Tarim have a continental warm temperate desert arid climate, with dry and windy weather and average annual precipitation ranging from 17.4 to 42.0 mm. The average annual evaporation is as high as 2500–3000 mm, making it one of the driest regions in China^30^.

The vegetation type in the Tarim’s lower reaches is mainly desert riparian. Accordingly, the plant community has low diversity and is mostly composed of species with high salt tolerance and drought resistance. There is also saline desert vegetation in localized areas with a single structure and poor species. Among the natural plants, the trees mainly include *Populus euphratica*, and the shrubs and herbs mainly include *Tamarix ramosissima, Tamarix hispida, Lycium ruthenicum, Alhagi sparsifolia, Apocynum venetu, Karelinia caspica, Glycyrrhiza inflata,* and *Phragmites communis*.

Since the 1960s, the Tarim River Basin has been under the influence of intensive human economic and social activities centered on the exploitation of soil and water resources. As a result, a 321 km stretch of the lower reaches of the Tarim River was cut off, the river’s terminal lakes (Lop Nor and Taitema Lake) dried up in 1970 and 1972, respectively, and the groundwater table dropped significantly^13^. Furthermore, in the Yengsu section of the river, the groundwater table depth is generally below 8 m, and even below 12 m in some sections. There has also been a precipitous decline in the region’s natural vegetation, such as *P. euphratica* and *Tamarix* spp. forest, accompanied by an increase in wind erosion and sanding and an intensification of land desertification. In short, the overall ecosystem has been severely damaged^30^, mainly by human activities. At the same time, the “Green Corridor” (it refers to the desert riparian forest ecosystem in the lower reaches of the Tarim River Basin, which is a verdant ecological corridor.) of the lower reaches of the Tarim, which is sandwiched between the Taklamakan and Quruq deserts, has shrunk dramatically and is on the verge of disappearing.

The increasing ecological problems and water crisis in the Tarim River Basin have become a hot research topic in relation to ecological and environmental problems in western China. In 2000, the Chinese government implemented an ecological water conveyance project aiming to raise groundwater levels, save the Green Corridor, and curb the manmade ecological deterioration^13^.

### Data acquisition

#### Plant sample monitoring

In order to understand the changes in groundwater table depth and surface vegetation during the water conveyance process, we established nine monitoring sections in 2000 (Fig. 6) along the lower reaches of the Tarim River water conveyance channel (the Qiwenkuoer River). The distance between each section is about 20 km. From the Daxihaizi reservoir downward, the monitoring sections are Akdun (I), Yahopmarhan (II), Yengsu (III), Abudali (IV), Karday (V), Tugmailai (VI), Argan (VII), Yiganbujima (VIII), and Korgan (IX). Among these, the spacing of the latter three cross-sections is 45 km (Fig. 6).

Fifty-five long-term ecological sample plots in total were constructed at the monitoring sections. Each sample plot is 50 m × 50 m and focuses on the number of individual plants as well as plant coverage, diameter at breast height, diameter at base, and the height and crown width of each tree and shrub. We surveyed the plant community in July every two or three years from 2000 to 2021. The plant sample monitoring was done using the methods described in Chen et al^5^. All of these methods were performed according to the relevant guidelines and regulations set forth by the Chinese government.

#### Groundwater table depth monitoring

Fifty-five groundwater monitoring wells (8 ~ 17 m depth) were installed near the sample sites of the nine monitoring sections to track the dynamic changes of groundwater table depth and water salinity during the water conveyance process. Initially, changes in the groundwater table depth were measured manually by conductivity. However, a HOBO water level logger (Campbell Scientific, Logan, UT, USA) was later installed at each monitoring well and record pressure-head readings (absolute pressure) were made every 18 h. Groundwater table depth from the soil surface is calculated using the corrected pressure-head measurement and the known sensor elevation.

#### Ecological water conveyance

The ecological water conveyance project of the lower reaches of the Tarim River was launched in May 2000. By 2020, 21 intermittent water conveyance channels had been implemented in the 321 km disconnected river channel of the lower reaches of the river. Except for the years 2006, 2007 and 2009, the river water reached the river's terminal, Taitema Lake, most of the time, delivering 84.45 × 10^8^ m^3^ of ecological water to the lower reaches of the river (Table 1). The total amount of groundwater and aeration zone recharged through river infiltration along the course of the river was about 30.6 × 10^8^ m^3^ and 42.15 × 10^8^ m^3^, respectively, and the amount of water entering Taitema Lake was about 11.7 × 10^8^ m^3^, accounting for 36.23%, 49.92% and 13.85% of total water delivered, respectively^31^.

**Table 1:** Information of ecological water conveyance in the lower reaches of Tarim River.

Water conveyance stage	Water conveyance date	Water discharge from Daxihaizi Reservoir (10^4^m^3^)	Where the water head reaches
1st	2000/5/14–7/12	9923	Karday
2nd	2000/5/14–12/31	22,655	30 km downstream Argan
3rd	2001/4/1–11/18	38,223	Taitema Lake
4th	2002/7/20–11/10	33,129	Taitema Lake
5th	2003/3/3–11/3	62,509	Taitema Lake
6th	2004/4/23–6/22	10,207	Taitema Lake
7th	2005/4/18–11/2	28,272	Taitema Lake
8th	2006/9/25–11/21	19,644	Korgan
9th	2007/10/10–10/21	1410	Karday
10th	2009/12/5–12/31	1066	Karday
11th	2010/6/25–11/11	36,393	Taitema Lake
12th	2011/1/7–11/23	85,211	Taitema Lake
13th	2012/4/27–11/27	66,716	Taitema Lake
14th	2013/4/25–11/5	48,769	Taitema Lake
15th	2014/6/17–6/26	727	172 km downstream Daxihaizi
16th	2015/8/18–11/5	46,218	Taitema Lake
17th	2016/8/11–10/31	67,611	Taitema Lake
18th	2017/4/27–12/31	121,461	Taitema Lake
19th	2018/2/26–11/21	70,006	Taitema Lake
20th	2019/8/11–12/31	46,352	Taitema Lake
21st	2020/9/5–11/9	28,084	Taitema Lake

### Methodology

#### Species diversity indices

These indices measure several different factors, such as number, evenness and richness. The Simpson diversity index reflects the species’ variation in number and biological diversity, the McIntosh index reflects the species’ evenness, and the Margalef index reflects their richness. In the sample site survey, the following methods were used in the calculations^31,32^.

$$ {\text{Simpson index}}:\lambda \user2{ = }{1}{ - }\sum\limits_{{i\user2{ = }1}}^{S} {p_{i}^{2} } $$

$$ {\text{McIntosh index}}:D\user2{ = }\left( {N - \sqrt {\left( {\sum {{\text{n}}_{i} } } \right)^{2} } } \right)/\left( {N - \sqrt N } \right) $$

$$ {\text{Margalef index}}:R = \left( {S - {1}} \right)/{\text{ln}}N $$

where *S* is the number of species, *N is* the total number of individuals of all plants, *A* is the sample area, n_i_ is the number of individuals of the *i-*th species, and *P*_*i*_ is the proportion of the multiplicity of the *i-*th species, i.e., the importance value. *P*_*i*_ = (relative density + relative cover + relative height)/3.

#### Vegetation coverage and density

Vegetation coverage denotes the percentage of the vertical projection of vegetation on the ground to the total area of the statistical area. Variations in vegetation coverage at the sample plot scale were calculated using the following method:

$$ {\text{Vegetation coverage}}:C = \sum\limits_{{i\user2{ = }1}}^{N} {a_{i} } /A \times 100{\text{\% }} $$

$$ {\text{Vegetation}}\;{\text{Density}}:D = N/A $$

where *N* is the total number of individuals of all plants, *A* is the area of the sample plot, and a_i_ is the canopy area of the *i*-th plant.

Additionally, using multi-source remote sensing MOD13Q1 and MCD12Q1 data with a temporal resolution of 16 d and a spatial resolution of 250 m, we calculated the basin-scale vegetation index (NDVI) and vegetation coverage changes since the implementation of the ecological water conveyance in the lower reaches of the Tarim River. To do so, we employed the Google Earth Engine (GEE) computing platform and multivariate statistical analysis to analyze changes in surface water area based on images from Landsat 5, 7 and 8 and ecological water conveyance data. Further, we applied the Ames Stanford Approach (CASA) model to estimate changes in NPP in natural vegetation, and the modified soil microbial respiration model R_H_ (Heterotrophic Respiration) to estimate the NEP (Net Ecosystem Productivity) of vegetation. Based on these techniques, were able to analyze the spatial variations in vegetation carbon source/sink.

## Data Availability

All of the data used in the present study are available from the corresponding author upon request.
